# Social jetlag and DXA-derived adiposity in Korean adults: A nationwide study using the 2024 Korea National Health and Nutrition Examination Survey

**DOI:** 10.1371/journal.pone.0362068

**Published:** 2026-11-05

**Authors:** Soojin Kim, Heewon Bae

**Affiliations:** 1 University of Texas Rio Grande Valley School of Medicine, Edinburg, Texas, United States of America; 2 Department of Neurology, Inje University Ilsan Paik Hospital, Goyang, Republic of Korea; University of Mississippi, UNITED STATES OF AMERICA

## Abstract

**Background:**

Social jetlag, the discrepancy between weekday and weekend sleep timing, is a behavioral marker of circadian timing mismatch and has been linked to obesity, mainly through conventional anthropometric measures. We examined its association with dual-energy X-ray absorptiometry (DXA)-derived adiposity using the nationally representative 2024 Korea National Health and Nutrition Examination Survey (KNHANES).

**Methods:**

This cross-sectional study included adults aged ≥40 years reporting usual weekday and weekend bedtimes and wake times. Social jetlag, the absolute difference between weekday and weekend sleep midpoints, was categorized as <1 h, 1– < 2 h, and ≥2 h, with ≥2 h as the primary exposure. The primary outcome was DXA-derived total body fat percentage; subtotal and trunk fat percentages were key secondary outcomes. Height, weight, and waist circumference were obtained by standardized KNHANES examination. Survey-weighted linear regression was adjusted for age, sex, height, average sleep duration, income, education, smoking, alcohol use, physical activity, hypertension, and diabetes.

**Results:**

Among 4,483 participants, 267 (6.0%) had social jetlag ≥2 h. In fully adjusted models, this group had higher total body fat percentage (β = 0.81 percentage points; P = 0.025), subtotal fat percentage (β = 0.86; P = 0.025), and trunk fat percentage (β = 1.01; P = 0.036), but not higher BMI or waist circumference. Associations persisted after adjustment for depressive symptoms, but were attenuated and no longer significant after adjustment for a chronotype-related proxy, and were not reproduced when social jetlag was corrected for sleep debt. Sleep duration variability showed no association.

**Conclusions:**

Social jetlag ≥2 h was associated with modestly higher DXA-derived total and trunk body fat percentage, differences not captured by BMI or waist circumference. Because the associations did not persist in chronotype-adjusted or sleep-corrected analyses, conventional social jetlag may partly reflect compensatory weekend sleep. These findings are hypothesis-generating and need longitudinal confirmation.

## Introduction

Social jetlag refers to the discrepancy in sleep timing between workdays and free days and reflects a misalignment between socially imposed schedules and individual biological timing [1,2]. In large-scale population surveys where workday and free-day sleep information is not directly available, weekday-weekend sleep timing differences are commonly used as a practical proxy for weekly sleep timing irregularity [3]. Unlike sleep duration, this measure captures differences in the timing of sleep across the week and may reflect repeated shifts in behavioral rhythms.

Sleep timing irregularity has been increasingly studied in relation to obesity and metabolic health. Previous observational studies and meta-analyses have reported associations between social jetlag and obesity-related outcomes, including BMI, waist circumference, overweight or obesity status, and weight change [4–6]. In Korea, a previous KNHANES-based study examined the association between weekday-weekend sleep timing mismatch and self-reported body weight change among adults [3]. These findings suggest that irregular sleep timing may be relevant to adiposity-related health outcomes in the general population.

Conventional anthropometric measures are widely used in clinical and epidemiological research, but they do not directly quantify body fat percentage or distinguish fat mass from lean mass. BMI may obscure differences in body composition, particularly across age and sex groups, while waist circumference is an indirect marker of central adiposity. Therefore, it remains uncertain whether weekday-weekend sleep timing mismatch is associated with directly measured adiposity rather than conventional measures of body size.

Dual-energy X-ray absorptiometry (DXA) provides a more direct assessment of body composition, including total and regional body fat percentage. In particular, DXA-derived trunk fat percentage may provide additional information on regional adiposity beyond BMI and waist circumference, although it should not be interpreted as a direct measure of visceral adipose tissue [7]. A prior study examining chronotype, social jetlag, and body composition in young adults suggested that sleep timing misalignment may be more strongly associated with fat distribution than with skeletal muscle mass [8]. However, evidence from nationally representative adult populations using DXA-derived adiposity measures remains limited.

The 2024 KNHANES includes weekday and weekend sleep timing data together with DXA-derived body composition measures. This provides an opportunity to evaluate whether weekly sleep timing irregularity is associated with directly measured adiposity in a nationally representative sample. Therefore, this study aimed to investigate the association between social jetlag, defined as weekday-weekend sleep midpoint discrepancy, and DXA-derived total, subtotal, and trunk body fat percentage in Korean adults. We also examined whether similar associations were observed for conventional anthropometric measures, including BMI and waist circumference.

## Materials and methods

### Study design and population

This cross-sectional study used data from the 2024 Korea National Health and Nutrition Examination Survey (KNHANES). KNHANES is a nationally representative survey of the non-institutionalized Korean population conducted by the Korea Disease Control and Prevention Agency using a complex, stratified, multistage probability sampling design [9]. Data are collected through standardized health interviews, health examinations, and nutrition surveys by trained personnel. In the 2024 survey cycle, whole-body DXA measurements were conducted in adults aged ≥40 years; accordingly, the present study focused on the DXA-eligible adult population. Because age is top-coded at 80 years in the publicly available KNHANES data, participants recorded as age 80 include adults aged 80 years or older. Older adults were retained to represent the full DXA-eligible adult population; potential age-related differences in sleep timing and adiposity were addressed through continuous adjustment for age and additional age-stratified analyses.

Of the 6,997 participants in the 2024 KNHANES, 4,680 were aged ≥40 years and were eligible for the present study. Of these, 197 were excluded because weekday or weekend bedtime/wake-time information was missing or invalid, leaving 4,483 participants with calculable social jetlag for descriptive analyses (Fig 1).

**Fig 1:**
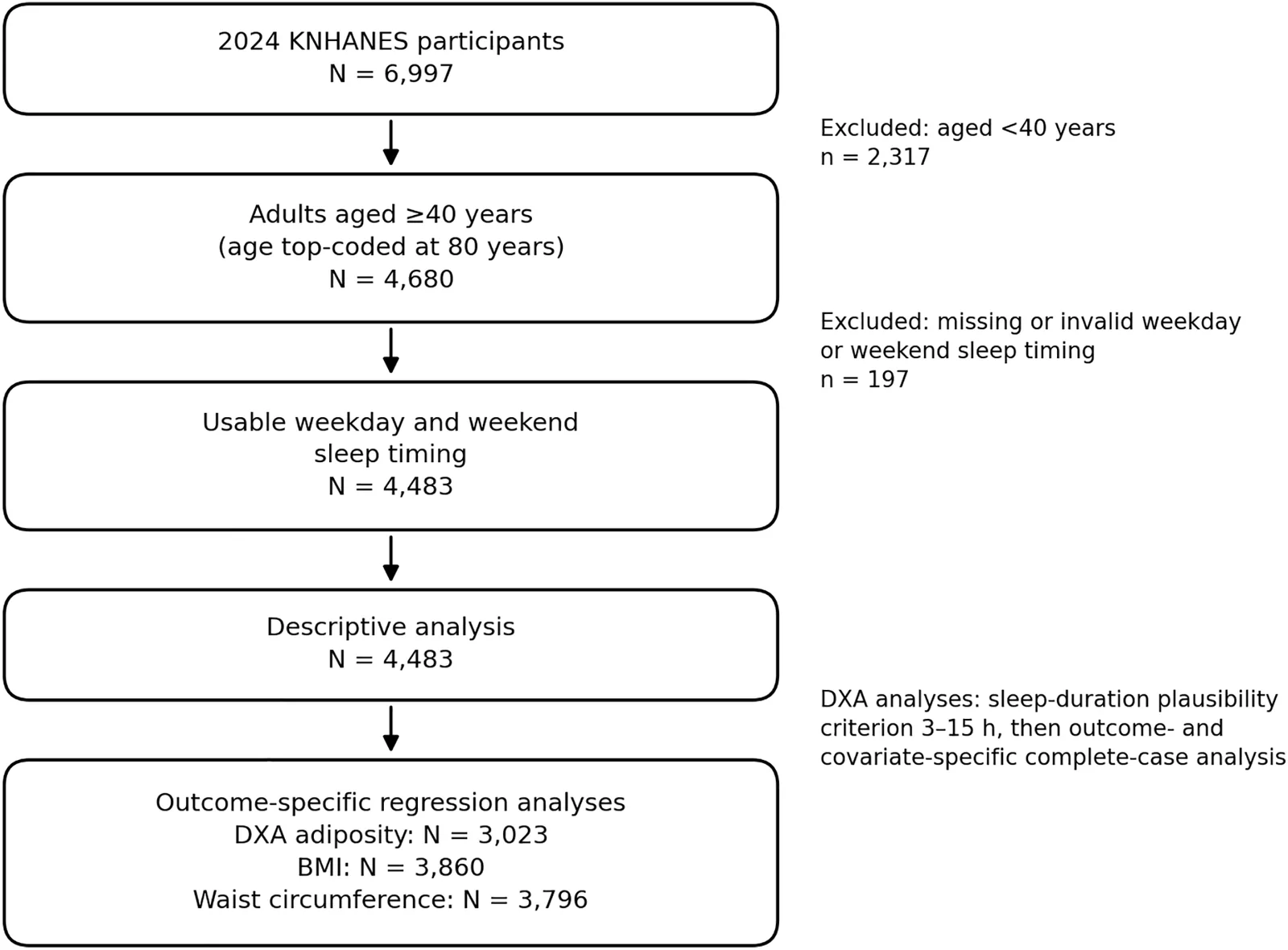
Participant flow diagram.

Flow of participants from the 2024 KNHANES source population to the descriptive and outcome-specific analytic samples.

The study protocol was reviewed and granted exemption by the Institutional Review Board of Inje University Ilsan Paik Hospital (IRB No. 2026-06-002). The KNHANES was conducted by the Korea Disease Control and Prevention Agency, and all participants provided informed consent at the time of survey participation. This study used publicly available, de-identified data and did not involve direct contact with participants.

### Assessment of sleep timing and social jetlag

Participants reported their usual bedtime and wake time separately for weekdays and weekends. Sleep duration was calculated as the elapsed time between bedtime and wake time, allowing for sleep periods that crossed midnight. Weekday and weekend sleep timing were analyzed as practical proxies for workday and free-day sleep timing because work schedule-specific information was not available for all participants; this approximation is widely applied in population surveys but does not correspond exactly to individually verified work and free days [10].

Sleep midpoint was calculated separately for weekdays and weekends as bedtime plus one-half of sleep duration. When calculated midpoints exceeded 24:00, they were converted to the corresponding time on a 24-hour clock. Social jetlag was defined as the absolute circular difference between weekday and weekend sleep midpoints, consistent with the conventional midpoint-based definition and prior KNHANES studies [2,3]. In addition, we derived sleep-corrected social jetlag (SJLsc) according to Jankowski [11]. For the common pattern of longer weekend sleep with an equal or later weekend sleep onset, SJLsc was defined as the absolute difference in sleep onset between weekends and weekdays; for the complementary pattern of longer weekday sleep with an equal or earlier weekday sleep offset, the absolute difference in sleep offset was used; otherwise, the conventional midpoint-based value was retained.

For descriptive analyses, social jetlag was categorized into three groups (<1 h, 1– < 2 h, and ≥2 h) to provide interpretable levels of increasing weekday–weekend timing discrepancy. Because no universally accepted clinical cutoff for social jetlag exists, these categories were used for epidemiologic interpretation rather than as validated clinical thresholds. The primary regression analysis used the originally specified contrast of social jetlag ≥2 h versus <2 h to focus on relatively marked timing mismatch. To reduce dependence on a single threshold, additional models treated social jetlag continuously and as a three-level categorical exposure using <1 h as the reference group.

Average sleep duration was calculated as [(weekday sleep duration × 5) + (weekend sleep duration × 2)]/7. Sleep duration variability was defined as the absolute difference between weekday and weekend sleep duration. A ≥ 2-h difference was evaluated as an exploratory threshold representing a relatively marked weekday–weekend discrepancy and facilitating comparison with the ≥ 2-h social jetlag analysis; it was not interpreted as a validated clinical cutoff. For the primary DXA regression analyses, weekday or weekend sleep durations <3 h or >15 h were considered implausible and excluded as a data-quality criterion. This criterion yielded a final DXA analytic sample of 3,023 participants.

### Assessment of adiposity and body composition

The primary outcome was DXA-derived total body fat percentage. Key secondary DXA-derived adiposity outcomes included subtotal body fat percentage and trunk fat percentage. In KNHANES 2024, whole-body composition was assessed in mobile examination units using a Horizon Wi DXA system (Hologic Inc., Waltham, MA, USA) by trained examination personnel following standardized protocols [12]. Subtotal body fat percentage was defined as body fat percentage excluding the head region, and trunk fat percentage was used as a regional adiposity marker. Because DXA-derived trunk fat percentage does not directly quantify visceral adipose tissue, it was interpreted as an indicator of regional adiposity rather than a direct measure of visceral fat.

Height and body weight were measured by trained staff using standardized KNHANES examination procedures, with participants wearing light clothing and no shoes; BMI was calculated as weight in kilograms divided by height in meters squared. Waist circumference was measured by trained staff to the nearest 0.1 cm at the midpoint between the lower rib margin and iliac crest with the participant standing after normal expiration. These anthropometric indices were evaluated as secondary outcomes to compare the associations of social jetlag with conventional body-size measures and directly measured adiposity.

Exploratory secondary analyses were performed to examine whether associations with social jetlag extended beyond percentage-based adiposity measures to absolute fat mass, muscle-related phenotypes, conventional obesity phenotypes, and recent weight change. These outcomes included DXA-derived fat mass (kg), low grip strength, low appendicular lean mass index, strict sarcopenia, abdominal obesity, and self-reported one-year weight change. Appendicular lean mass was calculated as the sum of lean mass in both arms and legs and divided by height squared to obtain appendicular lean mass index. Grip strength was defined as the maximum value among repeated right- and left-hand measurements. Low grip strength was defined as <28 kg in men and <18 kg in women, and low appendicular lean mass index as <7.0 kg/m² in men and <5.4 kg/m² in women [13]. One-year weight change was self-reported in KNHANES by comparison with body weight one year earlier; “no change” included changes of <3 kg, whereas weight gain and weight loss indicated changes of ≥3 kg in the respective direction.

### Covariates

Covariates were selected based on prior literature and clinical relevance [3–6]. The main models were adjusted for age, sex, height, average sleep duration, individual income quartile, education, current smoking, monthly alcohol use, aerobic physical activity, hypertension, and diabetes. Individual income quartile (incm) and education (educ) were modeled using the corresponding KNHANES categorical variables. Current smoking, monthly alcohol use, and aerobic physical activity were defined using the corresponding KNHANES indicator variables. Hypertension was defined from the KNHANES blood-pressure status variable, and diabetes was defined as the diabetes category of the HbA1c-based KNHANES diabetes-status variable. Height was included in DXA-derived adiposity models to account for residual differences in body size without directly adjusting for BMI; height was not included in models for BMI or waist circumference.

### Statistical analysis

All analyses accounted for the complex survey design of KNHANES using sampling weights, strata, and primary sampling units. Descriptive statistics in Table 1 are survey-weighted means for continuous variables and survey-weighted percentages for categorical variables, with unweighted sample sizes shown in the table headers. Overall differences across the three social-jetlag categories were evaluated using survey-weighted regression-based Wald tests. Survey-weighted linear regression models were used to evaluate the association between social jetlag and continuous adiposity outcomes, and survey-weighted logistic regression models were used for binary secondary outcomes; results are reported as β estimates or odds ratios with 95% confidence intervals.

**Table 1:** Baseline characteristics according to social jetlag category.

Characteristics	<1 h (n = 3,331)	1- < 2 h (n = 885)	≥2 h (n = 267)	P value
Age, years	61.1	53.5	50.8	<0.001
Average sleep duration, h	7.34	7.42	7.22	0.044
Weekday sleep duration, h	7.27	7.12	6.64	<0.001
Weekend sleep duration, h	7.50	8.18	8.68	<0.001
Social jetlag, h	0.21	1.28	2.93	<0.001
DXA total body fat, %	35.5	35.6	35.1	0.595
DXA trunk fat, %	37.0	36.7	36.8	0.589
BMI, kg/m²	24.3	24.4	24.8	0.182
Waist circumference, cm	85.3	84.4	86.1	0.113
Male sex, %	48.6	45.4	58.1	0.010
Current smoking, %	15.2	16.6	23.2	0.024
Monthly alcohol use, %	49.1	58.6	58.9	<0.001
Aerobic physical activity, %	38.8	42.6	39.7	0.283
Hypertension, %	60.5	50.2	50.5	<0.001
Diabetes, %	22.1	15.5	12.3	<0.001
One-year weight gain, %	18.2	20.4	27.5	0.009

Values are survey-weighted means or survey-weighted percentages; column sample sizes are unweighted. P values were obtained using survey-weighted regression-based Wald tests accounting for the KNHANES complex sampling design. DXA, dual-energy X-ray absorptiometry; BMI, body mass index.

Regression analyses were performed using outcome-specific complete-case samples for each outcome and covariate set. In the primary regression models, the analytic sample sizes were 3,023 for DXA-derived adiposity outcomes, 3,860 for BMI, and 3,796 for waist circumference.

Robustness analyses included modeling social jetlag continuously and as a three-level categorical exposure; additional adjustment for depressive symptoms using PHQ-8 (PHQ-9 total excluding the sleep item); evaluation of sleep-duration variability; analysis using sleep-corrected social jetlag; and an additional sensitivity analysis incorporating a sleep-timing-derived chronotype-related proxy. The proxy was the sleep-corrected midpoint of sleep on free days (MSFsc), approximated here using weekend sleep timing and calculated as the weekend sleep midpoint minus one-half of the excess of weekend sleep duration over weighted weekly average sleep duration when weekend sleep exceeded weekday sleep duration. Because MSFsc and social jetlag were derived from overlapping weekday/weekend sleep-timing information, MSFsc was not considered an independent chronotype measure or a conventional confounder in the primary model; it was evaluated only in a sensitivity analysis. Statistical significance was defined as a two-sided P value <0.05. All analyses were performed using R version 4.3.0 (R Foundation for Statistical Computing, Vienna, Austria), with the survey package used to account for the complex sampling design, including sampling weights, strata, and primary sampling units.

## Results

### Baseline characteristics

Among 4,483 adults aged ≥40 years with calculable social jetlag, 3,331 (74.3%) had social jetlag <1 h, 885 (19.7%) had social jetlag 1– < 2 h, and 267 (6.0%) had social jetlag ≥2 h (Table 1). Survey-weighted comparisons showed significant differences across social-jetlag categories in age (P < 0.001) and sex (P = 0.010).

Sleep characteristics differed across social-jetlag categories (Table 1). Participants with social jetlag ≥2 h had shorter weekday sleep duration and longer weekend sleep duration. Weekday sleep duration was 7.27 h in the < 1 h group and 6.64 h in the ≥ 2 h group, whereas weekend sleep duration was 7.50 h and 8.68 h, respectively (both P < 0.001). Average sleep duration also differed modestly across groups (P = 0.044).

Crude adiposity measures were generally similar across social-jetlag categories (Table 1). DXA total body fat percentage (P = 0.595), DXA trunk fat percentage (P = 0.589), BMI (P = 0.182), and waist circumference (P = 0.113) did not differ significantly across the three groups. Current smoking (P = 0.024), monthly alcohol use (P < 0.001), hypertension (P < 0.001), diabetes (P < 0.001), and one-year weight gain (P = 0.009) differed across categories.

### Association between social jetlag and adiposity

In survey-weighted multivariable linear regression models, social jetlag ≥2 h was associated with higher DXA-derived adiposity after adjustment for demographic, sleep, socioeconomic, behavioral, and cardiometabolic covariates. Compared with participants with social jetlag <2 h, those with social jetlag ≥2 h had higher total body fat percentage (β = 0.81 percentage points; 95% CI, 0.10 to 1.51; P = 0.025), subtotal body fat percentage (β = 0.86 percentage points; 95% CI, 0.11 to 1.61; P = 0.025), and trunk fat percentage (β = 1.01 percentage points; 95% CI, 0.07 to 1.95; P = 0.036). In contrast, social jetlag ≥2 h was not significantly associated with BMI (β = 0.08 kg/m²; 95% CI, −0.43 to 0.60; P = 0.748) or waist circumference (β = 0.51 cm; 95% CI, −0.95 to 1.96; P = 0.494) (Table 2).

**Table 2:** Association of social jetlag ≥2 h with adiposity measures.

Outcome	N	β	95% CI	P value
DXA total body fat, %	3,023	0.81	0.10 to 1.51	0.025
DXA subtotal body fat, %	3,023	0.86	0.11 to 1.61	0.025
DXA trunk fat, %	3,023	1.01	0.07 to 1.95	0.036
BMI, kg/m²	3,860	0.08	−0.43 to 0.60	0.748
Waist circumference, cm	3,796	0.51	−0.95 to 1.96	0.494

N represents the unweighted complete-case sample size for each model. β estimates and 95% confidence intervals were obtained from survey-weighted linear regression models. Models for DXA-derived adiposity outcomes were adjusted for age, sex, height, average sleep duration, individual income quartile, education, current smoking, monthly alcohol use, aerobic physical activity, hypertension, and diabetes. Models for BMI and waist circumference were adjusted for the same covariates except height. DXA, dual-energy X-ray absorptiometry; BMI, body mass index.

### Robustness analyses

When social jetlag was modeled continuously, each 1-hour increase was associated with higher DXA total body fat percentage (β = 0.25 percentage points; 95% CI, 0.01 to 0.49; P = 0.043), whereas the association with trunk fat percentage was positive but did not reach statistical significance (β = 0.28; 95% CI, −0.02 to 0.58; P = 0.063). In the three-category sensitivity analysis using <1 h as the reference, the 1– < 2 h group was not associated with total or trunk body fat percentage, whereas the ≥ 2 h group remained associated with higher total body fat percentage (β = 0.80 percentage points; 95% CI, 0.07 to 1.53; P = 0.031) and trunk fat percentage (β = 0.98; 95% CI, 0.02 to 1.94; P = 0.047). Additional adjustment for the MSFsc chronotype-related proxy attenuated the associations for social jetlag ≥2 h (total body fat: β = 0.61; 95% CI, −0.10 to 1.32; P = 0.089; trunk fat: β = 0.79; 95% CI, −0.15 to 1.74; P = 0.101). Sleep-corrected social jetlag analyzed continuously was not significantly associated with total body fat percentage (β = 0.14 per hour; 95% CI, −0.14 to 0.42; P = 0.328) or trunk fat percentage (β = 0.16; 95% CI, −0.19 to 0.51; P = 0.377). After additional adjustment for PHQ-8, associations remained significant for total body fat percentage (β = 0.81; 95% CI, 0.12 to 1.50; P = 0.022) and trunk fat percentage (β = 1.03; 95% CI, 0.10 to 1.95; P = 0.030). Sleep-duration variability ≥2 h was not significantly associated with total (β = 0.13; 95% CI, −0.48 to 0.73; P = 0.678) or trunk fat percentage (β = 0.22; 95% CI, −0.50 to 0.95; P = 0.543). These findings indicate that the main association was sensitive to related sleep-timing constructs and to correction for sleep debt (Table 3).

**Table 3:** Robustness and sensitivity analyses for social jetlag and DXA-derived adiposity outcomes.

Analysis	DXA total body fat, % β	95% CI	P value	DXA trunk fat, % β	95% CI	P value
Main model: SJL ≥ 2 h	0.81	0.10 to 1.51	0.025	1.01	0.07 to 1.95	0.036
SJL, per 1 h increase	0.25	0.01 to 0.49	0.043	0.28	−0.02 to 0.58	0.063
Three-category: 1– < 2 h vs < 1 h	−0.02	−0.52 to 0.49	0.951	−0.09	−0.70 to 0.52	0.762
Three-category: ≥ 2 h vs < 1 h	0.80	0.07 to 1.53	0.031	0.98	0.02 to 1.94	0.047
MSFsc-adjusted: SJL ≥ 2 h	0.61	−0.10 to 1.32	0.089	0.79	−0.15 to 1.74	0.101
Sleep-corrected SJL, per 1 h	0.14	−0.14 to 0.42	0.328	0.16	−0.19 to 0.51	0.377
PHQ-8 adjusted model	0.81	0.12 to 1.50	0.022	1.03	0.10 to 1.95	0.030
Sleep duration variability ≥2 h	0.13	−0.48 to 0.73	0.678	0.22	−0.50 to 0.95	0.543

Values are β estimates and 95% confidence intervals from survey-weighted linear regression models. Unless otherwise specified, models were adjusted for age, sex, height, average sleep duration, individual income quartile, education, current smoking, monthly alcohol use, aerobic physical activity, hypertension, and diabetes. The three-category analysis used <1 h as the reference. MSFsc denotes the sleep-corrected midpoint of weekend sleep used as a chronotype-related proxy. Sleep-corrected SJL was derived according to Jankowski [11]. PHQ-8 scores excluded the PHQ-9 sleep item. DXA, dual-energy X-ray absorptiometry; SJL, social jetlag.

### Subgroup and secondary analyses

In exploratory subgroup analyses, the association between social jetlag ≥2 h and DXA-derived adiposity appeared more evident among participants aged 40–64 years, particularly for trunk fat percentage. In this age group, social jetlag ≥2 h was associated with higher DXA trunk fat percentage (β = 1.13 percentage points; 95% CI, 0.13 to 2.13; P = 0.028) and with higher total body fat percentage (β = 0.88 percentage points; 95% CI, 0.13 to 1.63; P = 0.022). No statistically significant associations were observed among participants aged ≥65 years. In sex-stratified analyses, the direction of association was generally positive in both men and women, although the estimates were not statistically significant (S1 Table).

In selected secondary analyses, social jetlag ≥2 h was not significantly associated with any of the extended body-composition or weight-change outcomes examined, including DXA-derived total and trunk fat mass in kilograms, the highest tertile of DXA-derived trunk fat percentage, low grip strength, low appendicular lean mass index, strict sarcopenia, abdominal obesity, or self-reported one-year weight gain or weight loss (S2 Table). The higher crude prevalence of one-year weight gain in the ≥ 2 h group (Table 1) therefore did not persist after multivariable adjustment.

## Discussion

In this nationally representative sample of Korean adults aged ≥40 years, social jetlag was associated with modest but statistically significant differences in DXA-derived adiposity. Social jetlag ≥2 h was independently associated with modestly higher total body fat percentage, subtotal body fat percentage, and trunk fat percentage. In contrast, no significant associations were observed with BMI or waist circumference. When social jetlag was modeled continuously, the association remained significant for total body fat percentage but was attenuated for trunk fat percentage; associations with both outcomes remained significant after additional adjustment for depressive symptoms. Sleep duration variability was not significantly associated with DXA-derived adiposity. These findings suggest that social jetlag may be associated with differences in adiposity that are detectable by DXA but not by conventional anthropometric measures.

Previous studies have linked social jetlag to obesity and metabolic risk, including a recent systematic review and meta-analysis showing associations with several adiposity-related measures [4–6]. The present study extends this literature by focusing on directly measured DXA-derived body composition in a nationally representative adult population. Notably, crude fat percentages were similar across social jetlag categories, whereas adjusted models showed small positive associations. This pattern is plausible because participants with greater social jetlag were younger and more often men, characteristics generally associated with lower crude body fat percentage. Adjustment for demographic and other covariate differences therefore revealed a modest association that was not apparent from the unadjusted group means.

The association between social jetlag and adiposity may reflect an interaction between sleep timing, eating behavior, and circadian metabolism rather than a simple consequence of shorter sleep duration [5]. Weekly shifts in sleep timing can reorganize other behavioral rhythms, including meal timing, physical activity, and light exposure. Systematic reviews indicate that greater social jetlag and later chronotype are associated with less favorable dietary patterns, later eating, and greater irregularity in meal timing [14–16]. The related concept of ‘eating jetlag’ describes weekday-weekend variation in meal timing and has been associated with higher BMI in young adults even after consideration of social jetlag and chronotype [17]. These observations provide a plausible behavioral pathway through which irregular sleep timing may coincide with delayed or irregular energy intake and, over time, differences in adiposity.

A second potential mechanism involves circadian metabolism. Circadian misalignment may alter how the same behavioral exposure is processed metabolically. Experimental evidence suggests that misalignment of behavioral rhythms with internal circadian timing can impair insulin sensitivity and increase inflammatory markers independently of sleep loss [18]. These changes are relevant to adiposity because insulin sensitivity, glucose handling, and inflammatory signaling influence lipid storage, lipolysis, and adipokine secretion. Adipose tissue itself is also a circadian-regulated metabolic organ, and its rhythmic control of adipogenesis, lipolysis, inflammatory signaling, adipokine secretion, and thermogenesis may be disturbed when sleep-wake and eating rhythms are repeatedly shifted [19]. In this context, the modest differences in DXA-derived adiposity observed in our study may represent a population-level correlate of repeated behavioral timing mismatch, rather than direct evidence that social jetlag causes clinically apparent obesity.

Our findings are also consistent with prior body-composition research. Krueger et al. examined chronotype, social jetlag, and body composition among young adults and reported that larger social jetlag was associated with higher visceral fat mass, whereas skeletal muscle mass was not clearly associated with social jetlag [8]. Broader reviews of chronotype and body composition likewise suggest that later circadian preference is often accompanied by less favorable body-composition and dietary profiles [15,20]. Similarly, in our study, social jetlag was more consistently associated with adiposity than with muscle-related outcomes. This does not establish that social jetlag selectively affects fat tissue, but it supports further study of whether sleep-timing mismatch is more readily reflected in adiposity-related than muscle-related phenotypes in cross-sectional population data.

The association with trunk fat percentage is noteworthy but should be interpreted cautiously. DXA-derived trunk fat percentage is not a direct measure of visceral adipose tissue, but it provides a region-specific estimate of adiposity beyond BMI or body weight. In this study, the absolute difference in trunk fat percentage associated with social jetlag ≥2 h was modest. Therefore, the finding should not be interpreted as evidence of clinically meaningful central obesity at the individual level. Rather, it may indicate a subtle population-level adiposity signal related to sleep timing irregularity, which warrants further evaluation in longitudinal studies with direct measures of visceral adipose tissue.

Another important observation was that sleep duration variability was not significantly associated with DXA-derived adiposity, whereas conventional midpoint-based social jetlag showed positive associations with total and trunk fat percentage. However, the attenuation after adjustment for the MSFsc proxy and the null findings with sleep-corrected social jetlag indicate that the observed signal cannot be attributed exclusively to circadian timing mismatch. Conventional social jetlag may partly capture compensatory weekend sleep and weekday sleep debt in addition to timing irregularity. The present findings should therefore be interpreted as an association between weekday–weekend sleep-pattern mismatch and adiposity rather than as evidence of a specific causal circadian mechanism.

The pattern across age groups may also bear on the validity of the weekday–weekend approximation. The association with DXA-derived adiposity was more evident among participants aged 40–64 years and was not observed among those aged 65 years or older. Because weekday–weekend differences are most likely to reflect socially imposed timing constraints in individuals who follow conventional work schedules, this gradient is consistent with the proxy carrying more information in working-age adults. It also suggests that the present exposure definition may be least informative in older adults, in whom weekday and weekend timing are less likely to correspond to workdays and free days.

We did not observe significant associations between social jetlag and muscle-related phenotypes, including low grip strength, low appendicular lean mass index, and strict sarcopenia. These null findings do not contradict the main interpretation of the study. Rather, they suggest that the body composition signature of social jetlag may be more adiposity-specific than muscle-related in this population. The absence of significant associations with sarcopenic outcomes may also reflect limited event numbers, the cross-sectional design, or the need for longer-term exposure to detect muscle-related consequences.

This study has several strengths. First, it used a nationally representative sample and accounted for the complex survey design. Second, social jetlag was derived from weekday and weekend sleep timing, enabling evaluation of sleep timing irregularity beyond average sleep duration. Third, DXA-derived adiposity measures allowed direct assessment of body fat percentage and trunk fat percentage. Fourth, results were robust after adjustment for demographic, socioeconomic, lifestyle, sleep, metabolic, and depressive symptom variables.

Several limitations should be considered. First, the cross-sectional design precludes causal inference. Second, sleep timing was self-reported, and objective sleep measures such as actigraphy were not available. Third, direct questionnaire-based chronotype, shift-work schedule, meal timing, and light exposure were unavailable. Although MSFsc could be approximated from weekday/weekend sleep timing, weekday/weekend timing is not equivalent to individually verified work/free-day timing, and this approximation may be particularly imprecise in an older population in which a substantial proportion of participants may not follow conventional weekday work schedules. Moreover, MSFsc and social jetlag share underlying sleep-timing information; therefore, attenuation in the MSFsc-adjusted sensitivity analysis may reflect both chronotype-related variation and statistical overlap between the two constructs. Fourth, results were not robust when social jetlag was recalculated using the Jankowski sleep-corrected formulation, suggesting that sleep debt or compensatory weekend sleep may contribute to the observed conventional social-jetlag association. Fifth, crude DXA-derived fat percentages did not differ across social-jetlag categories, and the observed associations emerged only after multivariable adjustment, principally for age and sex; the findings are therefore dependent on model specification and should be interpreted with corresponding caution. Sixth, DXA-derived adiposity outcomes were available for 3,023 of the 4,483 participants with calculable social jetlag, and all regression analyses were restricted to complete cases; differential availability of DXA measurements or covariate information may have introduced selection bias. Seventh, multiple outcomes, subgroups, and sensitivity analyses were examined without formal adjustment for multiple comparisons, and the P values for the significant associations were close to the conventional threshold; the secondary and subgroup analyses should therefore be regarded as exploratory and hypothesis-generating. Finally, DXA-derived trunk fat percentage should not be interpreted as a direct measure of visceral adipose tissue, the observed effect sizes were modest, and residual confounding cannot be excluded.

In conclusion, conventional weekday–weekend social jetlag was associated with modestly higher DXA-derived total and trunk body fat percentage in Korean adults, whereas BMI and waist circumference showed no significant associations. The association was most evident for social jetlag ≥2 h but was attenuated in the MSFsc sensitivity analysis and was not reproduced using sleep-corrected social jetlag. These findings support a cautious interpretation that weekly sleep-pattern mismatch, potentially including both timing irregularity and compensatory sleep behavior, may be related to subtle adiposity differences. Longitudinal studies with objective sleep timing, verified work/free-day schedules, chronotype, meal timing, and direct visceral-fat measures are needed.

## Supporting information

S1 Table Association of social jetlag ≥2 h with key DXA-derived adiposity outcomes in sex- and age-stratified analyses. Values are β estimates with 95% confidence intervals and P values from survey-weighted linear regression models adjusted for the same covariates as the main model, except for the stratification variable where appropriate.
(DOCX)

S2 Table Association of social jetlag ≥2 h with selected secondary and extended body-composition outcomes. Values are β estimates for continuous outcomes and odds ratios for binary outcomes. Models were adjusted for the same covariates as the main model, with BMI included in binary phenotype models where appropriate.
(DOCX)
