# Supplementary material for: Social jetlag and DXA-derived adiposity in Korean adults: A nationwide study using the 2024 Korea National Health and Nutrition Examination Survey
Source: PLoS One. 2026 Nov 5;21(11):e0362068. doi: 10.1371/journal.pone.0362068 (PMC13739164; doi:10.1371/journal.pone.0362068)
Supplement: Supplementary file 1 [file pone.0362068.s001.docx]

**S1 Table. Association of social jetlag ≥2 h with key DXA-derived adiposity outcomes in sex- and age-stratified analyses**

| **Subgroup** | **N** | **SJL ≥2 h, n** | **DXA total body fat, % β (95% CI; P)** | **DXA trunk fat, % β (95% CI; P)** |
| --- | --- | --- | --- | --- |
| Men | 1,237 | 86 | 0.64 (-0.38 to 1.65; P = 0.216) | 0.65 (-0.61 to 1.91; P = 0.309) |
| Women | 1,786 | 85 | 0.65 (-0.42 to 1.72; P = 0.234) | 0.95 (-0.47 to 2.38; P = 0.189) |
| Age 40-64 years | 1,919 | 157 | 0.88 (0.13 to 1.63; P = 0.022) | 1.13 (0.13 to 2.13; P = 0.028) |
| Age ≥65 years | 1,104 | 14 | -0.48 (-2.47 to 1.50; P = 0.632) | -1.09 (-3.60 to 1.41; P = 0.390) |

Values are β estimates with 95% confidence intervals and P values from survey-weighted linear regression models adjusted for the same covariates as the main model (age, sex, height, average sleep duration, individual income quartile, education, current smoking, monthly alcohol use, aerobic physical activity, hypertension, and diabetes), except for the stratification variable where appropriate. SJL, social jetlag; DXA, dual-energy X-ray absorptiometry.
