# Supplementary material for: Social jetlag and DXA-derived adiposity in Korean adults: A nationwide study using the 2024 Korea National Health and Nutrition Examination Survey
Source: PLoS One. 2026 Nov 5;21(11):e0362068. doi: 10.1371/journal.pone.0362068 (PMC13739164; doi:10.1371/journal.pone.0362068)
Supplement: Supplementary file 2 [file pone.0362068.s002.docx]

**S2 Table. Association of social jetlag ≥2 h with selected secondary and extended body-composition outcomes**

| **Outcome** | **Type** | **N / events, n** | **Estimate (95% CI)** | **P value** |
| --- | --- | --- | --- | --- |
| DXA total fat mass, kg | β | 3,023 / -- | 0.74 (-0.24 to 1.72) | 0.138 |
| DXA trunk fat mass, kg | β | 3,023 / -- | 0.49 (-0.16 to 1.13) | 0.138 |
| High trunk fat %, top tertile | OR | 3,023 / 1,005 | 1.35 (0.84 to 2.15) | 0.215 |
| Low grip strength | OR | 3,640 / 237 | 1.55 (0.68 to 3.52) | 0.297 |
| Low appendicular lean mass index | OR | 3,023 / 1,330 | 1.37 (0.88 to 2.13) | 0.165 |
| Strict sarcopenia | OR | 2,879 / 109 | 2.19 (0.88 to 5.46) | 0.093 |
| Abdominal obesity | OR | 3,773 / 1,468 | 1.41 (0.84 to 2.39) | 0.194 |
| One-year weight gain | OR | 3,856 / 718 | 1.28 (0.92 to 1.77) | 0.146 |
| One-year weight loss | OR | 3,856 / 524 | 1.48 (0.95 to 2.30) | 0.079 |

Values are β estimates for continuous outcomes and odds ratios for binary outcomes, obtained from survey-weighted regression models adjusted for the same covariates as the main model, with BMI included in binary phenotype models where appropriate. DXA, dual-energy X-ray absorptiometry; BMI, body mass index; OR, odds ratio.
